# Damage-Associated Molecular Patterns and Th-Cell-Related Cytokines Released after Progressive Effort

**DOI:** 10.3390/jcm9030876

**Published:** 2020-03-23

**Authors:** Dorota Kostrzewa-Nowak, Andrzej Ciechanowicz, Jeremy S.C. Clark, Robert Nowak

**Affiliations:** 1 Centre for Human Structural and Functional Research, University of Szczecin, 17C Narutowicza St., 70-240 Szczecin, Poland; robert.nowak@usz.edu.pl; 2 Department of Clinical & Molecular Biochemistry, Pomeranian Medical University in Szczecin, 70-204 Szczecin, Poland; aciech@pum.edu.pl (A.C.);

**Keywords:** athletes, cytokines, damage-associated molecular patterns, progressive effort, soccer, T lymphocytes

## Abstract

Inflammation-induced processes commence with the activation of signalling pathways at the cellular level, which mobilize inflammatory cells and stimulate the secretion of chemokines, cytokines, and damage-associated molecular pattern molecules (DAMPs). Physical effort stimulates inflammation, contributing to muscle repair and regeneration. We have examined the impact of different protocols of progressive-effort tests on T-cell DAMP levels, extracellular cleavage products (fibronectin and hyaluronan), and Th-cell-related cytokine levels among soccer players. Thirty male soccer players with a median age of 17 (16–22) years performed different defined protocols for progressive exercise until exhaustion: (1) YO-YO intermittent recovery test level 1 (YYRL1, n = 10); (2) maximal multistage 20 m shuttle run (Beep, n = 10); and mechanical treadmill (MT, n = 10); and (3) shuttle-run test (n = 10). Blood samples were taken three times as follows: at baseline, post effort, and in recovery. Significantly higher post-effort concentrations of IL-4, IL-6, IL-10, and IFN-γ were observed in the Beep group, IL-4 in the YYRL1 group, and IL-6 and IFN-γ in the MT group as compared with the baseline values. Recovery values were significantly higher for concentrations of IL-4, IL-10, and IFN-γ in the YYRL1 group, only for IFN-γ in the Beep group, and for IL-6, IL-10, and INF-γ in the MT group as compared with the baseline values. Post-effort concentrations of DEFβ2, Hsp27, Fn, and UA in the Beep group and Hsp27 and HA in the YYRL1 group were significantly higher as compared with the baseline values. It seems the performed efficiency test protocols caused a short-term imbalance in Th1/Th2 cytokine levels without giving common molecular patterns. The rapidity of these changes was apparently related to specific physical movements and the type of running surface.

## 1. Introduction

Stress and physical effort are factors that significantly affect the functioning of the nervous system, endocrine adrenal function, and modulation of the immune system [1,2,3,4,5,6]. In response to various physiological states, the innate immune system can cause both T-cell activation and conversion of naïve T lymphocyte subsets into polarized effectors [1,7,8]. The immune response is critically affected by the type of physical effort, volume of training units, duration of the training program, training experience, diet, and other environmental factors, including the level of stress. The phenomenon of sterile inflammation, known to be a response to a psychological or a physical stressor, can also be an important mechanism involved in post-effort immune modulation [9,10].

Inflammation-induced processes occur with the activation of signalling pathways at the cellular level, which mobilize inflammatory cells and stimulate the secretion of chemokines, cytokines, and damage-associated molecular pattern molecules (DAMPs) [11,12]. It is well known that physical effort stimulates inflammation [13,14,15,16,17], which is a crucial mechanism contributing to muscle repair and regeneration [18,19,20]. Our previous studies have shown that progressive high-intensity effort performed by soccer players caused significant release of CD4^+^ T naïve cells into the circulation and indicated an important post-effort role for Th subsets [7,8]. Although there are numerous immunological studies describing the molecular mechanisms of immune responses to different antigens and pathogens, the impact of intensive physical effort on DAMP levels and extracellular signalling molecules is not yet fully understood.

Therefore, the main aim of our study was to examine the impact of different protocols of progressive-effort-until-exhaustion tests (YO-YO intermittent recovery test level 1 [21], an efficiency test on a mechanical treadmill, or a maximal multistage 20 m shuttle run test [22,23]) on T-cell DAMP levels, extracellular cleavage products (fibronectin and hyaluronan) and Th-cell-related cytokine levels among male professional soccer players.

## 2. Materials and Methods

### 2.1. Study Design

This was an experimental study performed at the Centre for Human Structural and Functional Research, University of Szczecin, Szczecin, Poland.

All participants belonged to the same sports club and took part in the same annual macrocycle training program. To analyze the impact of subsequent progressive effort on DAMP levels, extracellular cleavage products and Th-cell-related cytokine levels, measurements were taken before the test to assess baseline values (baseline), immediately after the progressive-effort- until-exhaustion test (post effort), and after around 17 hours of recovery time (recovery).

### 2.2. Participants

Thirty professional male soccer players, belonging to Pogoń Szczecin S.A. (aged 16 to 22 years, median age 17 years) and with at least five years of training experience, were recruited. All athletes qualified for this study played in a midfield position. Participants were divided into the following three groups who performed different protocols of the progressive-effort-until-exhaustion exercises: (1) the YYRL1 group with the YO-YO intermittent recovery test level 1 protocol [21]; (2) the Beep group with the maximal multistage 20 m shuttle run test [22,23]; and (3) MT group with efficiency test on a mechanical treadmill as described in our previous study [7,8].

Participants had no history of any metabolic syndrome (according to the International Diabetes Federation descriptions, i.e., without diabetes, prediabetes, abdominal obesity, high cholesterol, or high blood pressure) [24] or cardiovascular diseases (according to WHO definitions) [25]. They were nonsmokers and refrained from taking any medications or supplements known to affect metabolism. Participants were familiar with the exercise protocols as routinely used in the club. The recruitment process consisted of informing participants (and their parents, where appropriate) about the study and inviting them to take part by donating additional blood samples. All athletes who did not meet the inclusion criteria (e.g., by withholding consent or later withdrawing consent and goalkeepers) were excluded from the study. The study was approved by the Ethics Committee at the Regional Medical Chamber in Szczecin (no. 03/KB/VI/2017). Participants (and their parents, where appropriate) were fully informed of any risks and discomfort associated with the experimental procedures before giving their written consent to participate.

### 2.3. Progressive Test Protocols

All exercise tests evaluated the athlete’s aerobic capacity.

The YO-YO intermittent recovery test level 1 (YYRL1) consisted of 2 × 20 m shuttle runs at increasing speed (starting at 10 km/h) between audio signals. Each shuttle was separated by 10 s (2 × 5 m) of active recovery. Athletes performed the test until they were unable to reach the finish line before or at sound of the audio signal two times in a row. 

The maximal multistage 20 m shuttle run (Beep) test consisted of 20 m shuttle runs, without active recovery, at increasing speed (starting at 8.5 km/h). The test was performed until the athletes could not reach the finish line before or at the sound of the audio signal two times in a row.

The progressive efficiency test on a mechanical treadmill until exhaustion started with the 5 minutes of warm-up running with a speed of 5 km/h. During the main test the speed increased by 2 km/h after each 3 minutes of the test until exhaustion, i.e., until an athlete refused to run because of their maximal fatigue.

### 2.4. Methods

The following participants’ body mass and body composition parameters were determined: body mass index (BMI), basal metabolic rate (BMR), percentage of fat (FAT), fat free mass (FFM), and total body water (TBW), using a body composition analyzer (Tanita BC-418MA; Tanita, Tokyo, Japan). All participants then performed progressive-effort efficiency tests starting with 5 minutes of warm-up running at a speed of 5 km/h. 

Maximum oxygen uptake (VO2max) in the YYRL1 group was calculated according to the Bangsbo et al. formula [26] as follows:

$${\mathrm{VO}}_{2}\max\;\left(\mathrm{mL}/\min/\mathrm{kg}\right)=\mathrm{covered}\;\mathrm{distance}\;\left(m\right)\;\times \;0.0084+36.4$$

The VO2max in the Beep group was calculated according to Flouris at al. formula [27] as follows:

$${\mathrm{VO}}_{2}\max\;\left(\mathrm{mL}/\min/\mathrm{kg}\right)=\left(\max.\;\mathrm{attained}\;\mathrm{speed}\;\left(\mathrm{km}/h\right)\;\times \;6.65-35.8\right)\;\times \;0.95+0.182$$

Maximum oxygen uptake in the MT group was determined by using a state-of-the-art breath-by-breath gas exchange data analyzer (Quark CPET, Cosmed, Albano Laziale, Italy) [28].

Participant heart rates were monitored using the Garmin Forerunner 310XT (Garmin International, Inc., Olathe, KS, USA), and Garmin HRM3-SS soft straps. 

During the experiment, blood samples were obtained at the following three times from an elbow vein: before the test (pretest); no longer than 5 minutes after the test (post-test); and around 17 hours after the test, at the end of recovery time (recovery) (17 hours of recovery represented the usual mean period between two physical efforts, e.g., between a soccer match and the next training, or between two trainings). Blood samples were taken in tubes (7.5 mL S-Monovette tubes, SARSTEDT, Nümbrecht, Germany) with ethylenediaminetetraacetic acid (1.6 mg EDTA/ml blood, EDTA K3, SARSTEDT). 

White blood cell (WBC) and lymphocyte (LYM) counts were analyzed using a hematology analyzer (ABX Micros 60, Horiba ABX, Warsaw, Poland). 

The percentage of total lymphocytes and T-cells were determined using the expression of surface markers in erythrocyte-lysed blood samples, as described earlier [7]. All flow-cytometric analyses were performed using the BD Accuri™ C6 flow cytometer (Becton Dickinson, Franklin Lakes, NJ, USA) and the results of flow cytometric data were calculated using BD Accuri™ C6 software ver. 1.0.264.21. All enzyme-linked immunosorbent assays (ELISAs) were performed using a high throughput microplate reader Synergy H1 (BioTek Instruments, Inc., Vermont, USA).

For DAMPs (fibronectin (Fn); hyaluronic acid (HA); high mobility group protein 1 (HMG1); uric acid (UA) and cytokine (interleukin-2 (IL-2), interleukin-4 (IL-4), interleukin-6 (IL-6), and interleukin-10 (IL-10); and interferon gamma (IFN-γ)) level measurements, plasma samples were used. The IFN-γ/IL-4 ratio was calculated for every individual sample as a surrogate measure of the Th1/Th2 cytokine profile. In addition, all results of DAMPs and cytokine measurements in samples taken at the post-exercise time point or at the recovery time point were normalized to pre-exercise values and expressed as post-effort/baseline ratios or recovery/baseline ratios, respectively.

The T cells which were used to determine intracellular levels of DAMPs (defensing beta 2 (DEFβ2), heat shock 27 kDa protein 1 (Hsp27); thioredoxin (Trx)) were obtained using density gradient centrifugation in a lymphocyte-separation medium (Corning, Manassas, VA, USA) [29,30]. Separated cells were sorted using anti-human CD3^+^ antibody-conjugated magnetic nanoparticles with cell-separation magnets (BD IMag, BD Biosciences, San Jose, CA, USA) according to the manufacturer’s protocol. T-cells were lysed on ice (using ELISA lysis buffer 4, Cloud-Clone, Katy, TX, USA) according to the manufacturer’s protocol. 

DAMPs were assayed using appropriate ELISA kits according to the manufacturer’s protocols (Cloud-Clone). Only HA levels were measured using another assay (TECOmedical, AG, Sissach, Switzerland). The measurements of Th-cell-related cytokine (IL-2, IL-4, IL-6, IL-10, and IFN-γ) protein levels were performed using the BD Cytometric Bead Array (CBA) Human Inflammatory Cytokines Kit (BD Biosciences, San Jose, CA, USA) according to the manufacturer’s protocol, as described previously [7].

### 2.5. Statistical Analyses

All data are presented as medians (range). Statistical analyses were performed using Statistica version 13 (2017; TIBCO Software Inc., Palo Alto, CA, USA; http://statistica.io) using non-parametric tests. Whether differences observed among analyzed time points (pre-exercise vs. post-exercise vs. recovery) were significant was determined using Friedman’s analysis of variance for repeated measures followed by post-hoc Dunn tests with Bonferroni correction. Whether differences observed between analyzed groups (YYRL1 vs. Beep vs. MT) were significant was determined using Kruskal–Wallis analyses of ranks and median tests. The correlations between analyzed cytokines and DAMP molecules were assessed using Spearman’s rank correlation coefficient determination. The significance level used was p < 0.05.

## 3. Results

All three groups of participants (YYRL1, Beep, and MT) were homogenous according to basic characteristics (Table 1).

All individual data are included in Supplementary Materials Table S1. No significant differences in baseline values of WBC, LYM, T cells, IL-4, IL-10, DEFβ2, Hsp27, Fn, and HA, as well as in post-effort/baseline ratios for IL-10, Trx, HA, HMG1, and UA, and in recovery/baseline ratios for IL-2, IL-6, DEFβ2, Hsp27, Trx, HA, HMG1, and UA were found among the YYRL1, Beep, and MT groups as assessed by the Kruskal–Wallis test (Table 2, Table 3 and Table 4). There were significant differences in baseline values of IL-2, IL-6, IFN-γ, Trx, HMG1, and UA, as well as in post-effort/baseline ratios for IL-2, IL-4, IL-6, IFN-γ, and IFN-γ/IL-4, DEFβ2, Hsp27, and Fn, and in recovery/baseline ratios for IL-4, IL-10, IFN-γ, IFN-γ/IL-4, and Fn among the three analyzed groups (Table 3 and Table 4).

A significantly greater post-effort LYM count as compared with the baseline counts was found both in the Beep group and in the MT group. In addition, the post-effort WBC counts and LYM percentages in the latter group were greater as compared with baseline values. Significantly lower WBC and LYM counts, and a significantly lower percentages of T (CD3^+^) cells were observed at recovery time as compared with post-effort values both in the YYRL1 group and in the MT group. In addition, the LYM percentages at recovery time in the latter group were significantly greater as compared with at post effort (Table 2).

On the one hand, the post-effort concentrations of IL-4, IL-6, IL-10, and IFN-γ in the Beep group were significantly higher as compared with baseline values. Significantly higher post-effort concentrations of IL-4 were found in the YYRL1 group. On the other hand, the post-effort concentrations of IL-6 and IFN-γ in the MT group were higher as compared with baseline values. The IL-10 concentrations were significantly higher at recovery time as compared with post-effort values both in the YYRL1 group and in the MT group. In addition, IL-2 concentrations were significantly higher in the MT group and the IFN-γ concentrations in the YYRL1 group at recovery time. A comparison of baseline with recovery values revealed that the latter were significantly higher for concentrations of IL-4, IL-10, and IFN-γ in the YYRL1 group, only for IFN-γ in the Beep group, and for IL-6, IL-10, and IFN-γ in the MT group (Table 3).

On the one hand, post-effort concentrations of DEFβ2, Hsp27, Fn, and UA in the Beep group were significantly higher as compared with baseline values. On the other hand, post-effort concentrations of Hsp27, and also HA, were significantly higher in the YYRL1 group. Hsp27 concentrations were significantly lower in recovery time as compared with the post-effort values both in the YYRL1 group and in the Beep group. In addition, the HA concentrations at recovery time in the former group were also significantly lower as compared with the post-effort group. The Fn concentrations in recovery time were significantly higher only in the Beep group as compared with the baseline values (Table 4).

The analysis of correlations between concentrations of every cytokine with every DAMP was performed using Spearman rank correlation coefficient tests and all results are present in Supplementary Materials Table S2. The only significant correlations in the YYRL1 group were IL-10 x Fn, IL-4 x HA, IL-2 x HN, IL-2 x HMG1, IL-2 x UA, IL-6 x DEFβ2, and IFN-γ/IL-4 x HA for baseline measurements, and post-effort IL-2 x HA. In the Beep group, there were significant correlations for IL-4 x UA, IL-6 x HA, and IFN-γ/IL-4 x UA for baseline measurements; IFN-γ x Hsp27, IL-10 x DEFβ2, and IL-2 x UA at the post-effort time point; and IL-2 x HMG1, IL-6 x DEFβ2, IL-6 x Hsp27, IL-6 x Trx, and IL-6 x Fn at recovery. In the MT group, there were significant correlations for IL-4 x DEFβ2, IL-6 x Trx, IFN-γ/IL-4 x DEFβ2, and IFN-γ/IL-4 x Fn at pre-exercise time point; IL-10 x DEFβ2, IL-4 x DEFβ2, IL-4 x Hsp27, IL-6 x UA, and IFN-γ/IL-4 x Hsp27 at the post-effort time point; and IFN-γ x UA and IL-4 x HA at the recovery time point (Table S2).

## 4. Discussion

Exposure to acute stressors increases the concentrations of damage-associated molecular pattern molecules (DAMPs), inflammatory cytokines, and chemokines in the blood [10,31]. An important immunoprotective function of the acute stress response involves the expression of proteins that promote leucocyte trafficking and cytokine gene expression at sites of antigen entry [32,33]. The physical effort induced during progressive-effort-until-exhaustion tests is one possible stressor which could cause significant changes in the peripheral T-cell distribution [1,4,5,8]. Similar to previous reports [1,4,5,7,8], here, it was confirmed that the progressive effort caused significant increases in WBC and LYM counts, and changes in T-cell distribution (Table 2). It is noteworthy that the pattern of these changes was not related to the progressive-effort test protocol. In addition, the post-effort and recovery changes in cytokine levels differed between the groups analyzed. The YO-YO intermittent recovery test level 1, being the most typical type of exercise for soccer players [21], caused increases in both the anti- (IL-4, IL-10) and pro-inflammatory (IFN-γ) cytokines, while a more rapid response was observed after the maximal multistage 20 m shuttle run test.

Although the Beep test caused a significant increase in both pro-inflammatory IL-6 and INF-γ levels, comparable levels of anti-inflammatory IL-4 and IL-10 were observed. Both pro- (IL-6 and IFN-γ) and anti-inflammatory (IL-10) cytokine levels were higher as compared with baseline values after the test and after recovery in the MT group. These results indicate that the progressive effort induced by different protocols did not disturb the Th1- and Th2-related cytokine immunobalance. It seems that the most important cytokine in our observations was IL-10, which is known as a major anti-inflammatory cytokine responsible for inflammatory resolution processes and which inhibits initiation of pro-inflammatory protein synthesis [34]. The post-effort increases in cytokine levels are in line with literature data [8,10,15,17,32]. However, our study also indicated that the progressive effort probably did not trigger a general inflammation state in the soccer players.

A significant increase in DEFβ2 in the T-cells after the progressive-effort tests was observed only in the Beep group. There is very limited information regarding the identity of factor(s) that modulate DEFβ1 expression during Th1/Th2 homeostasis. It is well known that DEFβ1 expression depends on cell differentiation [35]. However, IL-1β remains the most potent agonist of TNF-α inducing DEFβ2 in some cells [36]. Our previous study gave evidence of no measurable changes in IL-1β [7,8]. Moreover, Pender at al. found no effects of T-helper type 2 (Th2) cytokine IL-4 on α-defensin expression [37]. Note that Pender at al. did not extend their study [37] to include the effects of anti-inflammatory cytokines such as IL-10, while we observed a significant increase in IL-10 in all studied groups.

It also appears that Hsp27 is an important DAMP protein related to protein molecular patterns generated following progressive effort. High glucose exacerbates the Hsp27 downregulation by pro-inflammatory cytokines [38] that could explain observed post-effort changes in T-cell levels of these proteins in the YYRL1 and Beep groups. Nahomi et al. gave evidence that a combination of pro-inflammatory cytokines downregulated Hsp27 and this response occurred only when all three cytokines (TNF-α, IL-1β, and IFN-γ) were present simultaneously, suggesting a synergistic cooperative mechanism in Hsp27 downregulation [38]. If our previous observations of a significant increase in TNF-α in a MT group [8] and the increase in IFN-γ noted in this study are considered, this could explain the significant change in Hsp27 levels observed in the MT group. An alternative explanation could be that this finding was related with the fact that the effort achieved on the mechanical treadmill used a lower friction force needed by the well-trained athletes, as these subjects know how to use the mechanical drive to reduce this force.

Although oxidative stress is a well described effect following physical effort [39,40,41,42] and the proteins related with this process, for example, Trx, are included as DAMP proteins [11], no significant changes in Trx were found in any of the studied groups. This requires further analysis, especially as the progressive effort causes a decrease in T-cell mitochondrial membrane potentials and DNA damage [43].

Local inflammation is initiated in the muscle tissue in response to progressive effort [13,14,16], which can influence T-cell differentiation and activation related to extracellular signalling molecules, for example, chemokines and cytokines [15,17]. Changes observed in T-cell subset distributions after the progressive effort [7,8,44] could have also manifested in the levels of extracellular DAMP proteins. HMG1 is a transcription factor which regulates both specific gene transcription and overall genomic stability in interactions with nucleotides, histones, transcription factors, and other chromosomal or nuclear proteins [45,46,47]. Increased levels of HMG1 were apparently not involved in extracellular post-effort responses in the studied groups. These findings confirm that although the progressive effort (especially on the mechanical treadmill) caused changes in pro- and anti-inflammatory cytokine levels, possibly the immune system global homeostasis was not imbalanced.

Interestingly, the levels of rapid (post-effort) immune responses seemed to be related to specific physical movements, as well as with the ground or deck used for the test. The most specialized movements were used in the YYRL1 group [26], where only the HA level rose during the experiment.

Fn levels were significantly higher after the test and after recovery as compared with the baseline values in the Beep group. The levels of studied extracellular DAMP proteins were higher than the baseline values in both groups which ran on the athletics track, while no changes were observed in the MT group where the participants ran on a mechanical treadmill. It is worth noting that the test used for the MT group was more similar to the Beep test than the YO-YO test, where the participants performed the exercise intermittent with recovery. It seems that the progressive effort induced on the athletic track caused the release of extracellular cleavage products such as fibronectin and hyaluronic acid. These DAMP proteins are probably related with imbalances in soft tissue caused by exhaustive effort and because of lower friction forces this imbalance was not observed in the MT group. However, this hypothesis needs further research.

The novelty of this study involves the assessment of the influence of three different protocols of progressive effort until exhaustion on the secretion of selected cytokines and DAMP molecules. To our knowledge, there are no studies which have examined varying protocols of this type of physical effort. Interestingly, the same state of participants’ exhaustion achieved by the different protocols (briefly, running back and forth without (Beep) or with (YO-YO) active recovery, and running on the mechanical treadmill, when an experienced athlete can use the treadmill drive to reduce effort) was not reflected at the molecular level.

Regarding the limitations of the study, there were small study groups because not all candidates met the inclusion criteria. The participants did not undergo any special microbiological examinations/infections. However, they were under the constant care of a club physician who would not allow infected individuals to take part in the study. Further analyses on larger groups including assessment of Th17 and Treg cytokines, and analyses of molecular mechanisms of differences observed between the analyzed groups, as well as including other types of physical effort, for example, resistance or explosive effort, are planned.

## 5. Conclusions

In conclusion, it must be stated that we did not observe similar molecular patterns related with progressive effort with the different progressive test protocols. It seems the efficiency tests performed by the soccer players caused a short-term imbalance in the Th1/Th2 cytokine levels. The rapidity of these changes seemed to be related to specific physical movements and type of running surface.

## Figures and Tables

**Table 1:** Characteristics and cardiorespiratory fitness measures of participants during the progressive-effort-until-exhaustion tests.

Variable	YYRL1 Group (*N* = 10)	Beep Group (*N* = 10)	MT Group (*N* = 10)	pKW ^1^
	Median (minimum – maximum)			
Age (years)	17 (16–20)	17 (16–22)	18 (16–21)	0.300
Height (m)	1.77 (1.67–1.89)	1.78 (1.67–1.84)	1.80 (1.60–1.89)	0.710
Mass ^2^ (kg)	67.4 (56.6–82.2)	72.3 (57.0–77.8)	69.8 (56.4–85.2)	0.818
BMI ^2^ (kg/m^2^)	21.6 (20.3–23.8)	22.5 (20.3–23.3)	21.8 (20.3–23.9)	0.717
FAT ^2^ (%)	9.8 (4.8–17.4)	9.2 (3.6–14.8)	7.9 (2.2–12.6)	0.818
FAT MASS ^2^ (kg)	6.3 (3.4–14.3)	6.7 (2.2–10.8)	5.5 (1.3–9.9)	0.963
FFM ^2^ (kg)	62.1 (49.8–72.1)	64.0 (52.7–69.2)	65.5 (52.3–75.3)	0.783
TBW ^2^ (kg)	45.5 (36.5–52.8)	46.9 (38.6–50.7)	48.0 (38.3–55.1)	0.672
Length of training experience (years)	10.0 (8.0–13.0)	10.5 (7.0–12.0)	11.0 (5.0–13.0)	0.863
Weekly training volume (hours)	12.0 (10.0–18.0)	11.8 (9.5–12.5)	12.0 (7.5–15.0)	0.169
HR_max_ (beats/min)	202 (194–212)	203 (197–217)	199 (178–210)	0.096
VO_2_max (mL/kg/min)	53.5 (46.5–58.6)	53.0 (48.3–57.8)	61.0 (55.8–67.3)	N.A.

^1^ Differences observed between analyzed groups (YYRL1 vs. Beep vs. MT) assessed using Kruskal–Wallis analysis of ranks. ^2^ Body mass and composition determined using a body composition analyzer (Tanita BC-418MA, Tanita, Tokyo, Japan). *N*, number of participants; BMI, body mass index; FAT, estimated lipid amounts, FFM, fat free mass; HRmax, maximum heart rate; TBW, total body water; VO2max, maximum oxygen uptake; YYRL1, YO-YO intermittent recovery test level 1 protocol (see methods); Beep, maximal multistage 20 m shuttle run test; MT, efficiency test on a mechanical treadmill; N.A., not applicable. No statistical comparison of VO_2_max values among groups was performed due to differing methods of determination.

**Table 2:** White blood cell (WBC), lymphocyte (LYM) counts, percentage of lymphocytes and T cells (CD3^+^) in studied participants’ blood samples.

Variable	YYRL1 Group (*N* = 10)				Beep Group (*N* = 10)				MT Group (*N* = 10)				pKW ^1^
	Baseline	Post-Effort	Recovery	p_F_	Baseline	Post-Effort	Recovery	p_F_	Baseline	Post-Effort	Recovery	p_F_	
	Median (Minimum–Maximum)				Median (Minimum–Maximum)				Median (Minimum–Maximum)				
WBC (10^9^/L)	5.3 (2.6–8.1)	7.8 (3.0–14.4)^B^	5.8 (3.1–10.9)	*0.025*	5.4 (4.0–8.1)	8.6 (4.8–12.9)	6.0 (3.9–8.4)	*0.149*	5.3 (4.0–6.4)^A^	8.8 (6.2–19.4)^B^	5.8 (3.7–8.9)	*0.003*	*0.630*
LYM (10^9^/L)	1.9 (1.2–3.3)	3.3 (1.4–6.1)^B^	1.9 (1.5–5.0)	*0.011*	1.9 (1.4–3.7) ^A^	3.2 (2.2–5.3)	1.6 (1.6–3.2)	*0.033*	2.2 (1.5–2.4)^A^	4.4 (2.4–4.9)^B^	2.5 (1.4–2.9)	*0.003*	*0.355*
LYM (%)	24.7 (10.4–35.5)	24.1 (14.0–35.4)	20.6 (15.0–32.9)	*0.606*	16.8 (9.6–20.8)	17.4 (12.0–23.0)	15.0 (11.3–22.2)	*0.905*	20.5 (12.2–24.6)^A^	29.6 (20.3–31.6)^B^	20.9 (12.1–26.8)	*< 0.001*	*0.064*
T cells (%)	74.8 (5.8–82.5)	69.9 (58.0–77.1)^B^	74.7 (64.0–82.2)	*0.021*	68.9 (58.5–81.7)	57.6 (39.6–75.5)^B^	75.6 (64.8–82.5)	*0.020*	65.4 (53.6–80.0)	58.7 (46.1–73.9)^B^	73.1 (60.2–80.8)	*< 0.001*	*0.051*

The analyses were performed before (baseline) and after the progressive effort (5 minutes post effort) and during recovery time (about 17 hours after the test). ^1^ Differences in baseline values among analyzed groups (YYRL1 vs. Beep vs. MT) were assessed using the Kruskal–Wallis analysis of ranks and the median test (pKW, Kruskal–Wallis p values). Significance levels of differences observed between analyzed time points (pre-exercise vs. post-exercise vs. recovery) were assessed using the Friedman’s analysis of variance for repeated measures (pF, Friedman’s ANOVA p values) followed by post-hoc Dunn’s test with Bonferroni correction. Post-hoc p values, ^A^ p < 0.05 for baseline vs. post-effort; ^B^ p < 0.05 for post-effort vs. recovery; ^C^ p < 0.05 for baseline vs. recovery. *N*, number of participants; Beep, maximal multistage 20 m shuttle run test; MT, efficiency test on mechanical treadmill; YYRL1, YO-YO intermittent recovery test level 1 protocol. For other abbreviations see Table 1.

**Table 3:** Median levels of interleukin-2 (IL-2), interleukin-4 (IL-4), interleukin-6 (IL-6), interleukin-10 (IL-10), and interferon gamma (IFN-γ) in studied participants’ plasma samples.

Variable	YYRL1 Group (N = 10)				Beep Group (N = 10)				MT Group (N = 10)				pKW ^2^
	Baseline	Post-Effort	Recovery	p_F_	Baseline	Post-Effort	Recovery	p_F_	Baseline	Post-Effort	Recovery	p_F_	
	Median (Minimum–Maximum)				Median (Minimum–Maximum)				Median (Minimum–Maximum)				
IL-2 (pg/mL)	2.7 (1.5–4.6)	2.6 (0.3–4.6)	4.6 (0.6–8.3)	0.061	1.5 (0.7–2.4)	3.9 (1.3–17.5)	1.2 (0.1–18.2)	0.067	5.9 (3.2–9.1)	3.3 (1.4–5.5)^B^	6.0 (2.3–8.6)	0.020	<0.001
ratio ^1^		1.1 (0.0–1.8)	1.8 (0.1–4.4)			3.8 (0.6–8.8)	0.8 (0.2–13.5)			0.6 (0.1–1.3)	1.0 (0.3–2.1)		<0.001 0.371
IL-4 (pg/mL)	1.2 (0.2–3.3)^A^	1.7 (1.3–4.5)	2.5 (1.2–5.3)^C^	0.001	1.4 (0.3–25.1)^A^	13.1 (7.2–27.8)	7.2 (2.4–15.8)	< 0.001	1.3 (0.5–2.5)	0.7 (0.3–1.5)	1.1 (0.4–2.4)	0.122	0.150
ratio		1.8 (0.0–7.9)	2.8 (1.2–7.9)			9.7 (0.3–23.1)	5.3 (0.1–15.2)			0.5 (0.1–2.4)	0.7 (0.4–2.4)		<0.001 0.002
IL-6 (pg/mL)	1.4 (0.4–4.6)	3.1 (1.3–7.9)	2.1 (1.3–7.9)	0.061	0.4 (0.1–20.6)^A^	13.1 (2.6–17.9)	2.1 (1.3–7.9)	0.002	1.3 (0.3–3.0)^A^	4.7 (2.3–6.2)	4.0 (2.2–9.2)^C^	0.002	0.039
ratio		2.3 (0.0–12.7)	1.3 (0.4–6.4)			34.0 (0.1–120.9)	5.5 (0.1–21.6)			2.9 (1.0–16.2)	3.5 (1.0–12.0)		0.004 0.075
IL-10 (pg/mL)	1.9 (0.8–6.6)	3.9 (2.0–11.9)^B^	11.2 (10.3–15.3)^C^	< 0.001	1.8 (0.9–3.8)^A^	5.9 (3.2–9.1)	3.3 (0.7–5.0)	0.007	1.5 (0.0–2.6)	1.9 (1.1–2.9)^B^	5.2 (3.0–13.4)^C^	< 0.001	0.191
ratio		1.9 (1.0–3.9)	5.5 (2.3–14.5)			3.0 (1.0–9.5)	1.7 (0.5–2.9)			1.4 (0.5–56.6)	3.6 (1.8–294.8)		0.163 <0.001
IFN- (pg/mL)	1.7 (1.1–10.5)	1.7 (0.1–6.4)^B^	13.5 (11.2–19.0)^C^	< 0.001	2.0 (1.1–15.2)^A^	6.8 (4.1–17.7)	6.3 (2.8–13.1)^C^	0.007	3.3 (2.4–4.5)^A^	5.0 (3.1–6.2)	12.9 (5.7–18.3)^C^	< 0.001	0.003
ratio		1.0 (0.0–1.3)	7.8 (1.2–15.4)			4.2 (0.3–14.1)	2.5 (0.5–9.7)			1.5 (1.0–2.4)	3.8 (1.5–7.7)		0.002 0.014
IFN-IL-4	1.8 (0.4–10.7)^A^	1.0 (0.0–5.1)	6.3 (2.1–13.7)	0.002	1.3 (0.6–6.1)	0.5 (0.2–1.9)	1.1 (0.2–3.2)	0.202	2.3 (1.2–4.5)^A^	8.2 (2.1–15.6)	11.0 (5.8–39.2)^C^	0.002	0.076
ratio		0.5 (0.0–3.0)	3.7 (0.2–7.2)			0.4 (0.1–2.3)	0.6 (0.1–5.3)			2.8 (0.9–12.9)	5.2 (1.4–12.2)		<0.001 0.010

^1^ Post-effort/baseline ratio or recovery/baseline ratio in “post-effort” column or “recovery” column, respectively. ^2^ Differences in baseline values or in ratio values among analyzed groups (YYRL1 vs. Beep vs. MT) were assessed using the Kruskal–Wallis analysis of ranks and the median test (pKW, Kruskal–Wallis p values for baseline values, post-effort/baseline ratio and for recovery/baseline ratio in upper row, middle row, or bottom row for each variable, respectively). For other abbreviations see Table 2.

**Table 4:** Median levels of T cell intracellular DAMPs (defensing beta 2 (DEFβ2), heat shock 27 kDa protein 1 (Hsp27), thioredoxin (Trx)) and extracellular DAMPs (fibronectin (Fn), hyaluronan (HA), high mobility group protein 1 (HMG1), uric acid (UA)) in studied participants’ blood samples.

Variable	YYRL1 Group (*N* = 10)				Beep Group (*N* = 10)				MT Group (*N* = 10)				pKW
	Baseline	Post-Effort	Recovery	p_F_	Baseline	Post-Effort	Recovery	p_F_	Baseline	Post-Effort	Recovery	p_F_	
	Median (Minimum–Maximum)				Median (Minimum–Maximum)				Median (Minimum–Maximum)				
DEF2 (pg/mL)	26.8 (10.8–30.0)	15.2 (10.8–29.8)	15.4 (10.9–29.8)	0.182	30.0 (10.1–44.3)^A^	58.9 (33.8–75.4)	57.0 (11.4–80.1)	0.032	30.8 (25.4–41.9)	25.9 (18.0–39.5)	24.0 (14.6–45.7)	0.061	0.077
ratio		0.8 (0.0–1.0)	0.7 (0.4–1.7)			1.7 (1.2–5.8)	1.9 (0.3–8.0)			0.8 (0.6–1.0)	0.7 (0.4–1.5)		<0.001 0.097
Hsp27 (ng/mL)	3.2 (2.4–4.7)^A^	4.1 (3.3–5.9)^B^	3.8 (2.4–4.9)	0.007	3.0 (2.2–4.3)^A^	3.7 (3.0–6.2)^B^	3.0 (1.6–4.4)	0.007	3.4 (2.7–4.0)	3.4 (2.1–4.5)	3.5 (2.8–4.5)	0.272	0.509
ratio		1.4 (1.0–1.9)	1.0 (0.8–2.1)			1.2 (0.9–2.5)	0.9 (0.5–1.9)			1.0 (0.6–1.2)	1.1 (0.8–1.4)		0.019 0.335
Trx (ng/mL)	21.3 (15.4–29.0)	25.1 (13.5–54.4)	28.2 (15.9–44.6)	0.082	30.3 (9.2–70.1)	17.4 (7.1–55.3)	26.3 (5.2–54.7)	0.272	10.6 (3.7–48.3)	15.9 (2.6–40.1)	21.8 (6.0–30.3)	0.670	0.034
ratio		1.1 (1.0–2.3)	1.5 (0.7–1.7)			0.7 (0.4–2.6)	0.7 (0.3–3.0)			1.7 (0.5–3.6)	1.8 (0.2–8.1)		0.071 0.164
Fn (mg/mL)	0.18 (0.16–0.22)	0.18 (0.06–0.23)	0.19 (0.11–0.22)	0.496	0.14 (0.06–0.35)^A^	0.21 (0.16–0.40)	0.22 (0.11–0.37)^C^	0.002	0.17 (0.05–0.38)	0.18 (0.05–0.33)	0.19 (0.09–0.36)	0.497	0.187
ratio		1.0 (0.0–1.2)	1.0 (0.6–1.2)			1.4 (1.1–3.9)	1.5 (0.6–4.0)			1.0 (0.6–1.3)	1.1 (0.7–2.5)		<0.001 0.017
HA (ng/mL)	19.3 (7.3–26.6)^A^	46.7 (23.2–59.3)^B^	18.6 (10.0–25.9)	< 0.001	12.4 (6.7–22.5)	23.9 (16.8–144.0)	12.3 (1.6–32.4)	0.061	17.3 (1.9–28.4)	18.4 (1.5–89.7)	24.7 (5.4–65.2)	0.150	0.284
ratio		2.7 (1.0–7.2)	1.0 (0.5–4.7)			1.8 (0.9–21.5)	1.2 (0.1–1.7)			1.0 (0.4–4.2)	1.9 (0.4–3.5)		0.122 0.083
HMG1 (ng/mL)	2.4 (1.2–4.1)	3.2 (1.5–9.4)	3.5 (1.1–8.4)	0.496	2.5 (1.9–3.0)	3.3 (2.3–10.7)	2.3 (1.4–9.6)	0.061	4.5 (3.5–5.0)	4.4 (3.7–5.6)	4.6 (2.9–8.4)	0.741	<0.001
ratio		1.3 (1.0–3.7)	1.4 (0.5–4.7)			1.4 (0.9–3.9)	1.0 (0.6–3.5)			1.0 (0.8–1.5)	1.1 (0.6–2.1)		0.074 0.903
UA (µmol/L)	261 (145–373)	282 (152–393)	289 (138–347)	0.067	345 (219–552)^A^	388 (278–571)	347 (210–527)	0.014	307 (241–383)	333 (211–392)	356 (260–402)	0.061	0.024
ratio		1.1 (1.0–1.3)	1.0 (0.8–1.2)			1.1 (1.0–1.3)	1.0 (0.6–1.6)			1.1 (0.9–1.2)	1.1 (0.9–1.4)		0.196 0.346

For abbreviations see Table 2 and Table 3.

## Supplementary Materials

The following are available online at https://www.mdpi.com/2077-0383/9/3/876/s1, Table S1: Raw data obtained for each individual participant during the study, Table S2: The results of correlation between analyzed cytokines and DAMP molecules analysis.
